# Beyond defense: microbial modifications of plant specialized metabolites alter and expand their ecological functions

**DOI:** 10.1111/nph.70254

**Published:** 2025-05-30

**Authors:** Kerstin Unger, Matthew T. Agler

**Affiliations:** ^1^ Plant Microbiosis Group, Institute of Microbiology Friedrich Schiller University Jena 07743 Jena Germany; ^2^ Cluster of Excellence Balance of the Microverse Friedrich Schiller University Jena 07743 Jena Germany

**Keywords:** defense, functional modification, microbiome, plant specialized metabolites, recruitment

## Abstract

Plant specialized metabolites (PSMs) are compounds that are not involved in primary metabolism but instead confer other roles for the plant host, often related to ecological interactions. In the field of plant–microbe interactions, many PSMs have traditionally been considered for their roles in shaping interactions with pathogens. However, it is increasingly clear that ‘defensive’ PSMs have broader functions in regulating assembly and functions of plant‐associated microbes, a phenomenon that is best studied in the rhizosphere. PSMs, however, are secreted throughout plants to mediate interactions with the environment. In this Tansley insight, we argue that these molecules also play outsize roles in shaping microbial community assembly and functions in the phyllosphere. Additionally, we argue that it is important to consider how microbial activity transforms PSMs, because this may shape how plants interact with the environment. Increased attention to these effects and improved strategies to understand them across scales will lead to insights into how microbial responses to PSMs shape broader plant interactions in the environment.

**Table:** 

	Contents	
	Summary	518
I.	Introduction	518
II.	PSMs have diverse functions at the plant–microbe interface	519
III.	PSMs can both deter and attract plant‐colonizing microbes	519
IV.	Microbes mediate PSM functions	521
V.	Microbial modification of PSMs manipulates plant ecology	522
VI.	Conclusion	524
	Acknowledgements	524
	References	524

## I. Introduction

The plant microbiome comprises diverse fungi, bacteria, viruses, oomycetes and protists. These microbes dynamically colonize practically all tissues throughout the life of plants from germination to death (Hassani *et al*., 2018). The plant microbiome as an entity, as well as individual microbes, has the potential to become imbalanced, so it is critical that plants keep colonizers in balance (T. Chen *et al*., 2020). To do so, the colonization of plant tissues by microbes is under control of a strict, multilayer system. Highly adapted pathogens that can easily invade plant tissues, for example, are under surveillance by intracellular effector receptors, while the growth and activity of opportunistic disease‐causing bacteria are kept in check by extracellular receptors and pattern‐triggered immunity (Pfeilmeier *et al*., 2021). In this way, the vast majority of microbial colonizers are maintained at the external surfaces of plants or in extracellular openings, for example, stomata or the apoplast.

Even though most colonizers are relegated to external tissues, they still form communities with structures and interactions driven by the host plant (Brachi *et al*., 2022). These communities strongly shape how plants communicate with their environments, for example by affecting plant growth, conferring resistance to abiotic stressors or supporting plant defense against pathogens (Shalev *et al*., 2022; Zheng *et al*., 2024). Important, largely open questions are: how are these loosely associated microbes managed?; and what are the mechanisms by which they shape plant functions? In this Tansley insight, we argue that plant specialized metabolites (PSMs) are a major tool for plants to manage not just pathogens but also their microbiomes and that in turn microbes modify the functions of PSMs. This broadening view of PSM–microbe interactions is already gaining importance in rhizosphere research (Huang *et al*., 2019; Voges *et al*., 2019; Thoenen *et al*., 2023). However, aboveground phyllosphere tissues also have massive PSM‐producing capacity and microbial colonizers that fundamentally shape plant health. Since the impact of PSMs with microbes in tissues like leaves is less well recognized, we place particular focus on examining the influence of known PSM–microbe interactions in the phyllosphere.

## II. PSMs have diverse functions at the plant–microbe interface

### 1. PSMs have complex ecological roles

In contrast to primary metabolites, such as sugars, proteins and fatty acids, PSMs are not part of the primary metabolism of plants but confer a specific function or play a specific role for the host. Plant specialized metabolites like phytohormones, for example, are important for the regulation of plant growth and development (Hauser *et al*., 2011), while others are important for defense and innate immunity (Ding *et al*., 2017). Generally, they fine‐tune a plant's response to environmental stressors (Moroldo *et al*., 2024) and overall mediate communication with microbes and other organisms (Trabelcy *et al*., 2023; Thoenen *et al*., 2024; Unger *et al*., 2024). There is no uniform classification system for PSMs; they can be grouped either based on their chemical structure (e.g. indoles, phenolics, N‐containing compounds and S‐containing compounds) or their biochemical biosynthesis pathway (e.g. phenylpropanoids and terpenoids). Regarding their effects on other organisms, defense metabolites are additionally classified according to the mode of their production and activation in the plant (phytoalexins, phytoanticipins and phytoavengins; Kliebenstein & Kvitko, 2023) or nondefensive compounds like phytohormones according to their function in communication (Dicke & Sabelis, 1988). Although the biochemical class can be related to a compound's mode of action, it is increasingly clear that this is not always true and the activity of individual PSMs is highly compound‐specific.

### 2. PSMs are secreted throughout plants at the plant–microbe interface

**Fig. 1:**
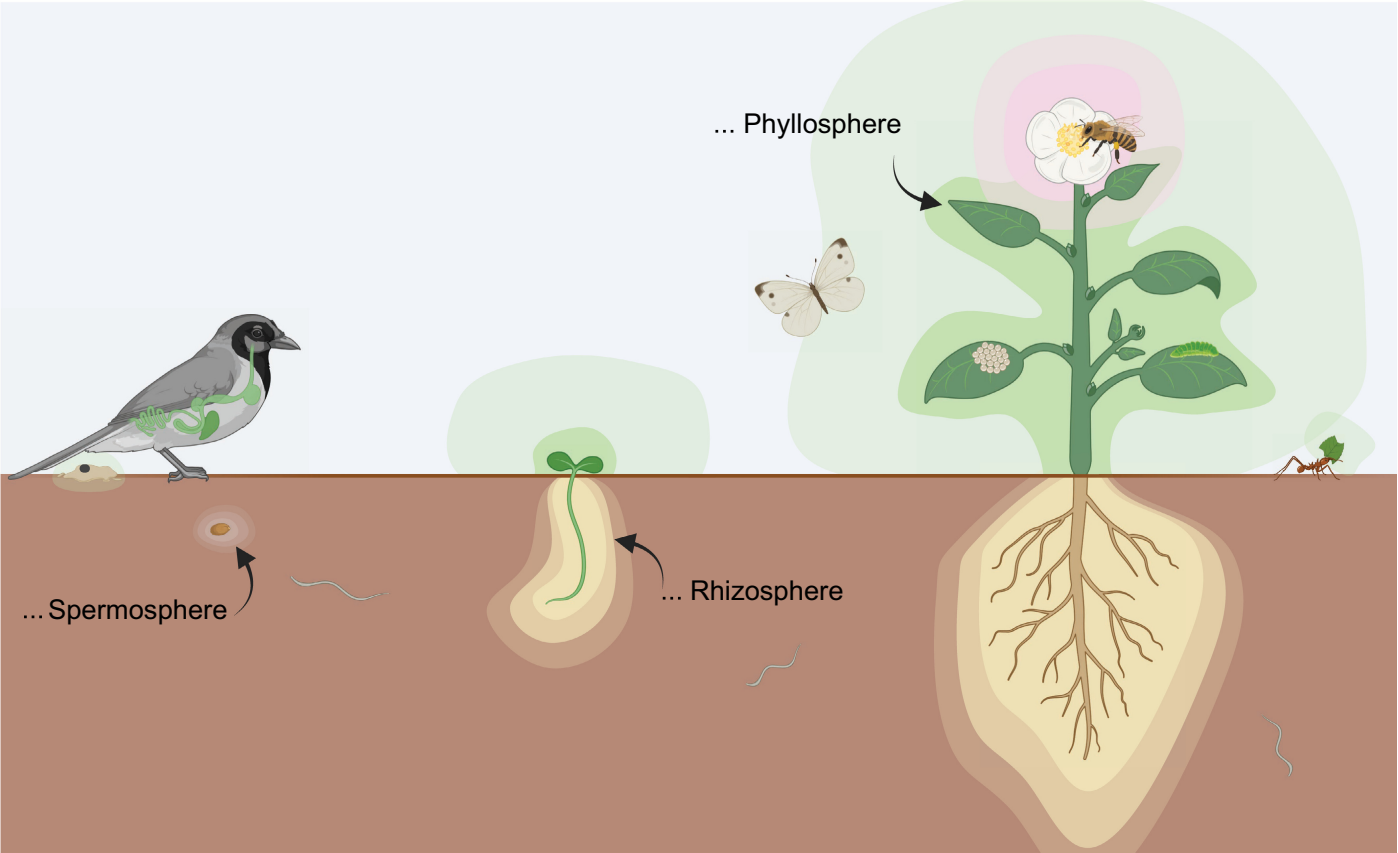
Plant specialized metabolites (PSMs) attract microbes to the spermosphere, rhizosphere and phyllosphere, including flowers. During its lifecycle, a plant exudes different PSMs in different organs. These PSMs make up the chemical landscape in the diverse plant‐associated habitats and influence microbial colonization. Around a germinating seedling, the zone of influence is rather small, whereas root PSMs are secreted further. In leaves, however, some PSMs are bound to the surface, whereas others are volatile and diffuse away in greater distances. PSMs and microbial modification of PSMs, which may happen inside or even outside of the plant system, have the potential to influence higher order interactions with insects and other animals. The figure was created in BioRender.com (https://BioRender.com/1sjz8xl).

Plants must interact with a broad diversity of organisms across both long distances and at the plant surface. This is managed in part by blanketing their organs with diverse PSMs (exudates) whose composition shift over time, space and in response to the environment itself. Plant specialized metabolite secretion begins at seedling germination (De‐la‐Cruz Chacón *et al*., 2013) and continues throughout the life of the plant (Fig. 1). Despite their functional differences, roots and leaves show remarkable parallels in PSM secretion. In roots, metabolite secretion tends to be cell‐type specific, especially along the length of the root (Kranawetter & Sumner, 2025). Cell‐type specificity is also observed in leaves, where glandular trichomes produce, store and release massive amounts of PSMs to the leaf surface (reviewed in Schuurink & Tissier, 2020), and PSMs are also produced and stored in epidermal cells and nonglandular trichomes (Gutiérrez‐Alcalá *et al*., 2000; Frerigmann *et al*., 2012). Plant specialized metabolites are ultimately released via combinations of both active and passive mechanisms. In both roots and leaves, localization is controlled by transporters of constitutively produced PSMs (Madsen *et al*., 2014; Xu *et al*., 2017) or targeted to specific locations in response to pathogens (Lanoue *et al*., 2010; Matern *et al*., 2019; Aryal *et al*., 2023; Liu *et al*., 2024). Subsequently, diffusion is probably critical to move PSMs from intercellular spaces to the environment, when small molecules pass along a concentration gradient out of cells. Diffusion depends strongly on moisture in precipitation, microbial biofilms, cuticle water pores and stomatal water films (extensively reviewed in Ossola & Farmer, 2024). This could create a major difference between roots where moisture and microbial density are relatively uniform and leaves where both are highly patchily distributed (see also Box 1). Although nonvolatile root PSMs can diffuse into soil, they are far more concentrated at the root surface (Okutani *et al*., 2020) so that long‐distance effects in both compartments are probably mostly mediated by volatiles (Delory *et al*., 2016). Regardless of transport mechanisms, the external PSM layer is bound to be a primary site of interaction with microbes which arrive at the plant surface via soil, air deposition or other sources. Indeed, this helps explain why abundances of PSMs are correlated to abundances of both pathogenic and nonpathogenic microbes (Gaube *et al*., 2023) and underscores the importance of understanding their roles across multiple scales (Box 1).

Box 1 **Scale matters for microbial interactions with plant specialized metabolites (PSMs) in leaves**

Scaled to the size of a human, an average leaf would be nearly 5000 km^2^ (Remus‐Emsermann & Schlechter, 2018). Given that leaf microbes are patchily distributed (Schlechter & Remus‐Emsermann, 2025), the interaction zone of individual bacterial cells is likely limited to their neighborhood. Indeed, microbial interactions have been measured to span up to 20 μm, whereas interaction with leaf trichomes can reach as far as 100 μm (Esser *et al*., 2015). We may therefore expect that PSMs only influence leaf microbes when they happen to be localized in the interaction zone of a microbe. Current sampling methods usually involve crushing whole leaves to extract microbial DNA and metabolites (Gaube *et al*., 2023), but this may produce misleading correlations between PSMs and microbes for two reasons. First, we might miss small‐scale interactions in such bulk results. Second, we might infer interactions with intracellular PSMs, which are usually not in contact with microbes in a living leaf. For a more accurate picture of interactions in the phyllosphere, Armitage *et al*. (2023) postulate adjusting the experimental designs to the scale of the research question. Supporting the importance of this approach, the spatial resolution of bacterial and fungal diversity together with plant gene expression in leaf cross‐sections provided a first glimpse into spatially resolved interactions (Saarenpää *et al*., 2024). This suggested local host regulation of microbial hotspots by plant immunity. In addition, direct MS imaging techniques may be valuable, especially to acquire spatial resolution of surface‐deposited PSMs, which are in contact with microbes in living tissues (reviewed in García‐Rojas *et al*., 2024). Combining both techniques has the potential to more precisely study how PSMs influence microbes in leaves and vice versa. Although this is currently difficult because of limited methods to link PSM distribution to the distribution of microbial cells, correlative approaches combining FISH and MALDI‐MS imaging are improving (Bourceau *et al*., 2023) and may soon be applicable in tissues like leaves. Thus, although few correlative approaches have been applied in leaves, it will be critical to transfer advances from other systems to plants.

## III. PSMs can both deter and attract plant‐colonizing microbes

### 1. PSMs deter nonhost pathogenic microbes

One important function of many PSMs is to provide broad resistance against nonadapted plant pathogens. Plants therefore secrete compounds, including terpenoids, phenylpropanoids and alkaloids, among others, which function as a nonhost defense strategy. To name just a few examples of antimicrobial PSMs in leaves, the monoterpene linalool inhibits *Xanthomonas oryzae* pv *oryzae*, the causal agent of rice bacterial blight (Taniguchi *et al*., 2014). *Arabidopsis thaliana*, petunia and tomato leaves produce increased amounts of phenylpropanoids like ferulate or p‐coumarate, which reduces *Botrytis cinerea* infections (Oliva *et al*., 2020). Similarly, glucosinolate breakdown products like isothiocyanates decrease the growth and virulence of diverse bacterial and fungal pathogens (Fan *et al*., 2011; J. Chen *et al*., 2020). Generally, the inhibition profiles of PSMs are specific and not generalizable for entire biochemical classes (reviewed in Kliebenstein, 2024).

Besides growth inhibition, PSMs can also affect the activity of microbes, which may influence microbe–microbe or plant–microbe interactions. The glucosinolate breakdown product 4‐methylsulfinylbutyl isothiocyanate (4MSOB‐isothiocyanate), for example, directly inhibits the expression of virulence proteins in bacterial pathogens (Wang *et al*., 2020). *In vitro*, isothiocyanates also have the potential to interfere with quorum sensing (Bendary *et al*., 2024). Similarly, plant phenolic volatiles like carvacrol and eugenol inhibit quorum sensing in soft‐rot causing *Pectobacterium* spp., decreasing virulence (Joshi *et al*., 2016). Thus, PSMs have diverse defensive effects that help protect plants from nonhost pathogenic microbes.

### 2. PSMs recruit adapted microbes to plants

Although PSMs in relation to microbes are most often studied for defensive effects, they can also serve as nutrient or energy sources (Flagan & Leadbetter, 2006; Thoenen *et al*., 2024; Unger *et al*., 2024). In the rhizosphere, bacteria with specialized gene clusters catabolize saponins like α‐tomatine in the tomato rhizosphere (Nakayasu *et al*., 2023), triterpenes, such as thalianin, thalianyl fatty acid esters and arabidin, exuded from *A. thaliana* roots (Huang *et al*., 2019) or isoflavones in the soybean rhizosphere (Aoki *et al*., 2024). In leaves, recent studies in *A. thaliana* and wheat have shown strong links of aliphatic glucosinolates and benzoxazinoids, respectively, to specific recruitment patterns in leaf bacteriomes (Brachi *et al*., 2022; Unger *et al*., 2024; Xiang *et al*., 2024). Indeed, glucosinolates are the basis for a metabolic network of leaf‐associated bacteria, which can degrade these PSMs as sole carbon sources (Unger *et al*., 2024). Although it has not been shown yet in leaves, rhizosphere bacterial isolates can utilize DIMBOA‐Glc as well as its breakdown products, MBOA, as carbon sources (Thoenen *et al*., 2024) and such activity could explain leaf recruitment patterns as well. These activities can help explain why it seems at first counterintuitive that the same PSMs may both deter nonhost pathogens and recruit commensals.

## IV. Microbes mediate PSM functions

**Fig. 2:**
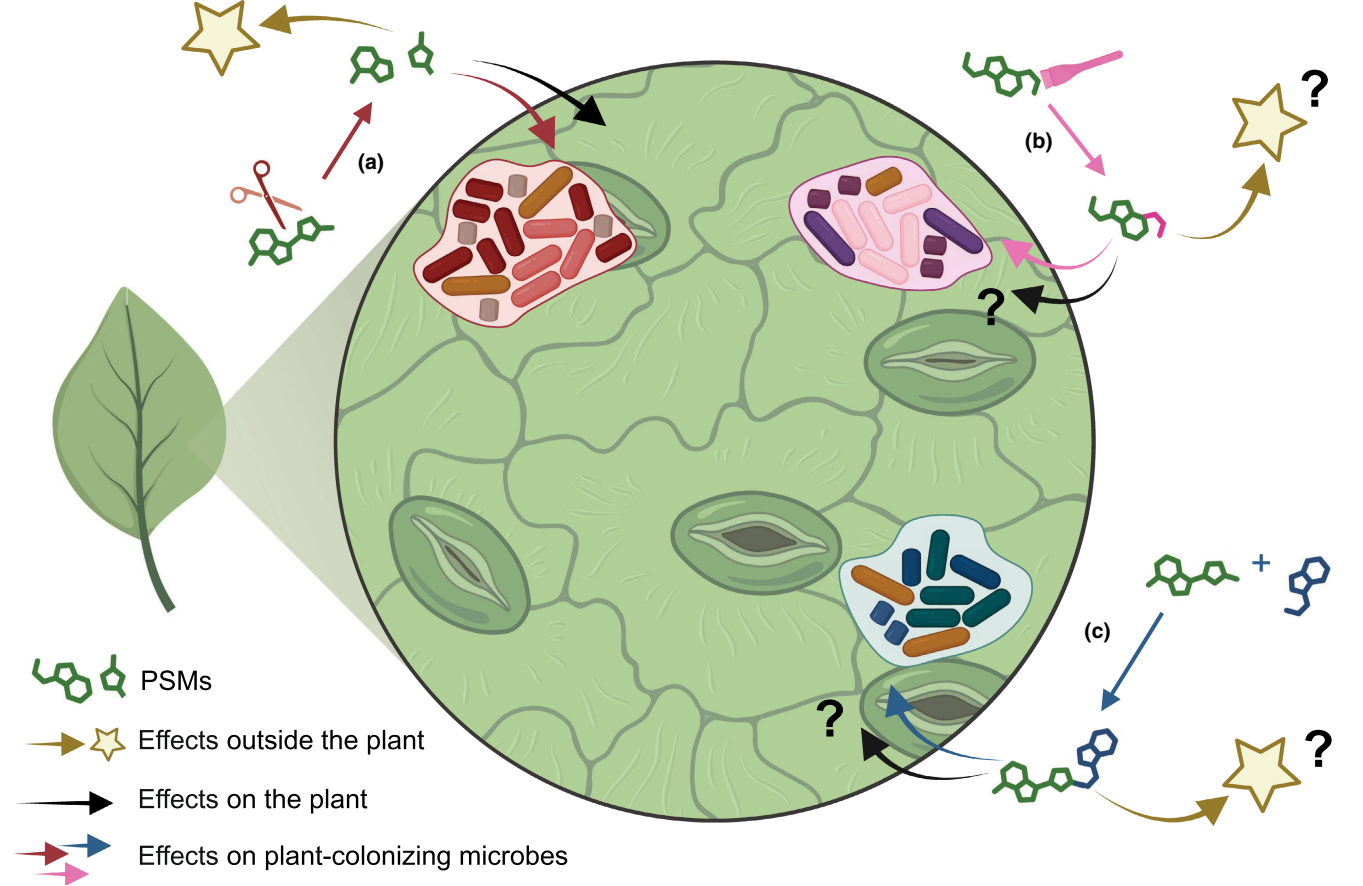
Modifications of plant specialized metabolites (PSMs) by plant‐associated microbes have diverse effects. Leaves exude PSMs (green metabolites), which are modified by microbes in multiple ways. (a) The red bacteria can degrade (scissors) PSMs. (b) The pink bacteria can chemically transform (paint brush) PSMs. (c) The blue bacteria can modify PSMs by coupling them to their own metabolites (blue metabolites). All these modifications may go along with either loss‐ or gain‐of‐function compared with the original PSMs, which may have effects on the plant host, the PSM‐modifying microbes, other co‐colonizing microbes, or they may even have broader implications on the interaction of the plant with its environment (stars). Proposed effects are marked with question marks. The figure was created in BioRender.com (https://BioRender.com/m72r332).

While correlations between microbes and chemical landscapes in leaves can reflect direct selection as described above, they may also reflect microbial alteration of chemical signatures. Stallforth *et al*. (2023) group possible modifications of metabolites into degradation, alteration and expansion (Fig. 2), resulting in loss‐of‐function (PSM rendered inactive) or gain‐of‐function (change in or creation of PSM activity). For leaf PSMs, most modifications with clear functional relevance are degradations (Fig. 2a). A few examples of transformations (Fig. 2b) and molecular expansion (Fig. 2c) are known, but functional relevance is usually less well described. Examples are the oxidation of root‐exuded thaliandiol to 3‐keto‐thaliandiol by *Agaromyces* sp. (Huang *et al*., 2019) and the oxidation of various sesquiterpenes by root‐associated bacteria in the sweetgrass *Vetiveria zizanioides* (Del Giudice *et al*., 2008). Generally, both gain and loss‐of‐function modifications are described in either the phyllosphere or rhizosphere, but many probably have relevance in both compartments. Thus, future research is needed to elucidate modifications and their functional relevance. Here, we outline what is currently known.

### 1. Loss of PSM function modifications

The detoxification of defensive PSMs represents a classical loss‐of‐function modification where PSMs lose their defensive function because of microbial modifications. Modifications commonly arise as resistance mechanisms against PSMs and are widespread in leaf colonizing microbes. Two major mechanisms are ubiquitous: detoxification by degradation or by transformation of the PSM. In a well‐studied example, bacterial pathogens express the hydrolase SaxA to detoxify isothiocyanates, which are glucosinolate breakdown products, by hydrolyzing them to corresponding amines (Fan *et al*., 2011; van den Bosch *et al*., 2020; Fig. 2a). This activity facilitates higher colonization and virulence in Brassicaceae plants (Fan *et al*., 2011; van den Bosch *et al*., 2020; J. Chen *et al*., 2020). The fungus *Sclerotinia sclerotiorum* similarly detoxifies isothiocyanates by relying on both degradation via a SaxA homolog (Fig. 2a) and glutathione conjugation (Fig. 2c), which contributes to its resistance, especially when SaxA is absent (J. Chen *et al*., 2020). The degradation of the antimicrobial isoflavone daidzein in the soybean rhizosphere is mediated by a specific gene cluster in *Variovorax* sp. starting with a hydroxylation of daidzein to 8‐hydroxy‐daidzein (Fig. 2b) and subsequent breakdown of the ring structure (Aoki *et al*., 2024). Other loss‐of‐function cases are known due to changes in metabolite profiles. For example, in flowers, volatile PSM profiles can be affected by both bacteria and fungi, with changes that can negatively affect bee visitation (Rering *et al*., 2018; Cellini *et al*., 2019). In leaves, the composition of microbiomes also shows important correlations to detectable volatile organic compounds (Gaube *et al*., 2023). Although the functional implications are not well understood, this could alter the roles that volatile PSMs play in attraction, for example in oviposition (Whiteman & Peláez, 2021).

### 2. Gain‐of‐function modifications

A gain‐of‐function is when PSMs alterations create new metabolites with functions that did not exist originally. For example, microbes associated with plants can elicit drastic changes in volatile profiles that attract insects. The citrus pathogen *Candidatus Liberibacter asiaticus* alters citrus volatiles, which causes its vector, the aphid *Diaphorina citri*, to more often choose infected plants (Mann *et al*., 2012). Controlled inoculation of grapes with microbes can significantly alter their volatile emissions. While some microbes appear to have a repellent effect, volatiles contributed by yeast fungi, in particular, increased preference for grapevine moth oviposition (Tasin *et al*., 2018). In the rhizosphere, plants modify soil properties by exudation of PSMs, resulting in soil feedbacks on other plants. For example, in maize, the benzoxazinoid breakdown product MBOA alters the rhizosphere microbiome, which activates defense in subsequent plants (Hu *et al*., 2018). Benzoxazinoids can also alleviate negative soil feedbacks from other plants (Gfeller *et al*., 2024). Microbes are critical for both effects, probably in part because they break down glycosylated benzoxazinoids like DIMBOA‐Glc to toxic compounds like (M)BOA (Thoenen *et al*., 2023, 2024). In general, deglycosylation, primarily by plant glucosidases, activates diverse defensive PSMs, such as glucosinolates, isoflavones or coumarins (Husebye *et al*., 2002; Suzuki *et al*., 2006; Stringlis *et al*., 2018). Microbial glucosidases have also been shown to break down these PSMs (Gaya *et al*., 2017; Deflandre *et al*., 2022; Unger *et al*., 2024). Thus, we propose that catabolism of PSMs by bacteria, especially by hydrolysis of glycosylated molecules, broadly contributes to gain of defensive functions (Fig. 2a; Box 2).

Box 2 **Evolutionary processes shaping plant specialized metabolite (PSM) interactions in the plant–microbe holobiont**

The holobiont concept describes a host and microbes whose association can determine phenotypes and ecology (Bordenstein & The Holobiont Biology Network, 2024). Because a holobiont can potentially be subject to selection as a whole, it has been suggested that specialized interactions of beneficial plant‐colonizing microbes with PSMs could arise via coevolutionary processes (Mesny *et al*., 2023). Coevolution describes the process by which two organisms directly drive one another's adaptation, known for example to drive gain, loss and diversification of genes underlying virulence of obligate pathogens and resistance of plant hosts (Kemen *et al*., 2015). Plants have evolved a strong dependence on microbes for protection (Durán *et al*., 2018), and this can depend on PSMs (Zhong *et al*., 2022). Thus, PSM–microbe interactions could evolve to shape these protective interactions. Establishing this causality is difficult and would require showing clear evidence of strong selection on the chemical interactions that could drive coevolution. Exploring this idea is important, however, because it could revise thinking about diversification of PSMs, which is largely thought to occur directly in response to threats like herbivory (Speed *et al*., 2015).

**Fig. B1:**
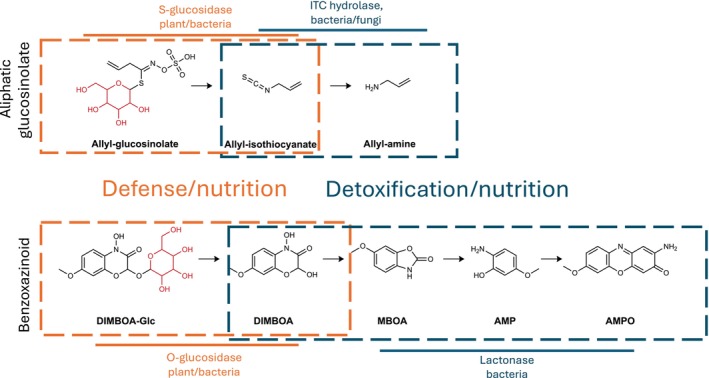
Different plant specialized metabolites (PSMs) produced by the Brassicaceae plants and Poaceae grasses that have convergently evolved functions in defense are also metabolized in very similar ways by plant‐associated microbes. AMP, 2‐amino‐5‐methoxyphenol; AMPO, 2‐amino‐7‐methoxy‐phenoxazin‐3‐one; DIMBOA, 2,4‐dihydroxy‐7‐methoxy‐1,4‐benzoxazin‐3‐one; ITC, isothiocyanate; MBOA, 6‐methoxybenzoxazolin‐2(3H)‐one.

Until now, few mechanisms of protective microbe‐PSM interactions have been resolved (Zhong *et al*., 2022), a prerequisite to understanding their evolution. One promising candidate is bacterial deglycosylation of PSMs, which can release products with new properties, such as pathogen inhibition (as mentioned in the ‘Gain‐of‐function modifications’ section). For glucosinolates and benzoxazinoids, the processes of bacterial deglycosylation and further modifications are remarkably convergent (Fig. B1), which may suggest functional relevance. In particular, the deglycosylation step, which is catalyzed by microbial glucosidases, can release compounds that may protect plants. Indeed, for benzoxazinoids, at least some of the benefits for host plants appear to depend on microbes (Gfeller *et al*., 2024). If so, it should be simple to design experiments to test on the host side if certain benefits of gain, loss or diversification of glycosylated PSMs depend on the presence of bacteria that can transform them. Similarly, experiments could test whether bacteria benefit by coevolving traits specialized for the glycosylated PSM diversity of their host plant. In testing for coevolution, it is important to keep in mind the null hypothesis that coevolution may not always be necessary. While some bacterial glucosidases show substrate specificity and may need to adapt to new substrates (Cebeci *et al*., 2022), others have extremely wide substrate spectra (Deflandre *et al*., 2022). In other words, bacterial protection of plants by PSM transformation can be an emergent trait of the holobiont but does not directly imply coevolution. In general, thorough studies on topics like functional effects of metabolite modifications and enzyme diversity, all required to make conclusions, are still needed.

## V. Microbial modification of PSMs manipulates plant ecology

Brakhage (2024) recently put forward the conceptually useful idea that certain metabolites produced by microbes function as ‘hub’ compounds, disproportionately affecting surrounding organisms. Plant specialized metabolites also have ecological implications far beyond the producer plant, so that they fit the description of hub compounds. Interestingly, in many cases, it is microbes outside of the plant that shape these distant effects. Perhaps best studied are the roles of bacteria in herbivorous insects. Diverse insect species take advantage of bacterial detoxification of plant PSMs to help them cope with a herbivorous lifestyle (Shukla & Beran, 2020). In a more complicated interaction, bacteria help protect *Leucoagaricus gongylophorus*, the fungus reared by leaf‐cutter ants, by breaking down toxic PSMs in the leaves that the ants harvest and feed to the fungus (Francoeur *et al*., 2020). Adaptations to deal with plant PSMs also strongly affect interactions with other animals. For example, human consumption of cooked brassica vegetables introduces intact glucosinolates to the intestines. Gut bacteria that express myrosinases release isothiocyanates, which are thought to positively influence human health (Liou *et al*., 2020). Bacteria that detoxify plant PSMs may even help stabilize plant–animal symbioses. When the bird *Pycnonotus xanthopygos* eats fruits of *Ochradenus baccatus* shrubs, they simultaneously take up *Pantoea* sp. The bacteria help the bird cope by detoxifying isothiocyanates released from the seeds during digestion. Subsequently, the bacteria increase the germination success of seeds that pass through the digestive tract, helping the plant to propagate (Trabelcy *et al*., 2023).

## VI. Conclusion

The multiple, context‐dependent ecological roles of PSMs are already illustrated by the various classification systems that exist for them. Increasingly, it is also clear that as secreted molecules, they are embedded at the forefront of plant–microbe–environment interactions. These interactions can involve reciprocal modifications of PSMs that inevitably influence how other organisms react – be it the host plant, animals or the microbes themselves. This concept has so far been less well accepted in the phyllosphere than, for example, the rhizosphere. However, phyllosphere organs like leaves host diverse microbial communities while prolifically synthesizing, storing and secreting PSMs that have well‐described functions in defense, attraction and allelopathy. Thus, it is important that we take a more holistic perspective that includes implications of broader interaction partners, not just defense against pathogens. To better understand these complex, multilayered interactions, there is a need to develop new tools like spatially resolved methods to detect microbes and PSMs in parallel (Box 1). We expect such approaches to increase mechanistic insights into host plant selection of microbiomes, as well as their functions, potentially leading to new applications.

## Competing interests

None declared.

## Disclaimer

The New Phytologist Foundation remains neutral with regard to jurisdictional claims in maps and in any institutional affiliations.
